# Risk Factors and Predictors of Severe Leptospirosis in New Caledonia

**DOI:** 10.1371/journal.pntd.0001991

**Published:** 2013-01-10

**Authors:** Sarah Tubiana, Marc Mikulski, Jérôme Becam, Flore Lacassin, Patrick Lefèvre, Ann-Claire Gourinat, Cyrille Goarant, Eric D'Ortenzio

**Affiliations:** 1 Unité d'Epidémiologie des Maladies Infectieuses, Institut Pasteur de Nouvelle-Calédonie, Réseau International des Instituts Pasteur, Noumea, New Caledonia; 2 Service de Réanimation, Centre Hospitalier Territorial de Noumea, Noumea, New Caledonia; 3 Laboratoire de Recherche en Bactériologie, Institut Pasteur de Nouvelle-Calédonie, Réseau International des Instituts Pasteur, Noumea, New Caledonia; 4 Service de Médecine Interne, Centre Hospitalier Territorial de Noumea, Noumea, New Caledonia; 5 Service de Médecine, Centre Hospitalier du Nord, Koumac, New Caledonia; 6 Laboratoire de Sérologie-Virologie, Institut Pasteur de Nouvelle-Calédonie, Réseau International des Instituts Pasteur, Noumea, New Caledonia; David Geffen School of Medicine at UCLA, United States of America

## Abstract

**Background:**

Leptospirosis is a major public health concern in New Caledonia (NC) and in other tropical countries. Severe manifestations of the disease are estimated to occur in 5–15% of all human infections worldwide and factors associated with these forms are poorly understood. Our objectives were to identify risk factors and predictors of severe forms of leptospirosis in adults.

**Methods and Findings:**

We conducted a retrospective case-control study of inpatients with laboratory-confirmed leptospirosis who were admitted to two public hospitals in NC in 2008–2011. Cases were patients with fatal or severe leptospirosis, as determined by clinical criteria. This approach was meant to be pragmatic and to reflect the routine medical management of patients. Controls were defined as patients hospitalized for milder leptospirosis. Risk and prognostic factors were identified by multivariate logistic regression. Among the 176 patients enrolled in the study, 71 had criteria of severity including 10 deaths (Case Fatality Rate = 14.1%). Three risk factors were independently associated with severe leptospirosis: current cigarette smoking (OR = 2.94 [CI 1.45–5.96]); delays >2 days between the onset of symptoms and the initiation of antibiotherapy (OR = 2.78 [CI 1.31–5.91]); and *Leptospira interrogans* serogroup Icterohaemorrhagiae as the infecting strain (OR = 2.79 [CI 1.26–6.18]). The following post-admission laboratory results correlated with poor prognoses: platelet count ≤50,000/µL (OR = 6.36 [CI 1.79–22.62]), serum creatinine >200 mM (OR = 5.86 [CI 1.61–21.27]), serum lactate >2.5 mM (OR = 5.14 [CI 1.57–16.87]), serum amylase >250 UI/L (OR = 4.66 [CI 1.39–15.69]) and leptospiremia >1000 leptospires/mL (OR = 4.31 [CI 1.17–15.92]).

**Conclusions:**

To assess the risk of developing severe leptospirosis, our study illustrates the benefit for clinicians to have: i) the identification of the infective strain, ii) a critical threshold of qPCR-determined leptospiremia and iii) early laboratory results. In New Caledonia, preventative measures should focus on early presumptive antibacterial therapy and on rodent (reservoir of Icterohaemorrhagiae serogroup) control.

## Author Summary

Leptospirosis is a neglected tropical disease and a public health concern worldwide. Factors responsible for the progression towards severe forms have not been clearly established. However, pathogen- as well as host-related factors are both believed to play a role in the development of severe leptospirosis. This study aimed to determine risk and prognostic factors independently associated with severe leptospirosis in laboratory-confirmed cases in adults in New Caledonia. Our study provides important results on these factors. One major finding was the independent association between the serogroup Icterohaemorrhagiae and severe leptospirosis in the multivariate analysis. Though empirically recognized, we think that this association between this highly prevalent serogroup and most severe forms of the disease was seldom (if ever) clearly demonstrated. Our data also illustrate the benefit of using a critical threshold of qPCR-determined leptospiremia to assess the risk of developing severe leptospirosis in patients after their admission to hospital.

## Introduction

Leptospirosis is a major threat to public health but little is known about the actual disease burden, and consequently, the disease has been neglected. Nevertheless, it is recognized as the most widespread zoonosis worldwide and an important and possibly emerging infectious disease. It occurs mostly in tropical and subtropical areas where conditions for transmission are favorable but is also known to occur in temperate climates [1], [2]. Leptospirosis has also emerged as a disease of the adventure traveler, especially those participating in water sports [3].

According to estimates from the World Health Organization, more than 500,000 severe cases occur every year worldwide. In New Caledonia (NC) and other French overseas tropical or sub-tropical territories (French Caribbean, French Guyana, Polynesia and Reunion Island), leptospirosis is a significant public health concern [4]. In NC, leptospirosis is known to be endemic with epidemic bursts occurring during hot rainy periods (from December to March) [5], [6]. The average annual incidence is 45 cases per 100,000 inhabitants (2006–2009) but can reach 150 per 100,000 inhabitants during the rainiest months.

The spectrum of symptoms is extremely broad and leptospirosis shares common clinical signs with many acute febrile diseases, such as influenza, dengue fever or malaria. Severe manifestations occur in 5–15% of human infections and are typified as: i) Weil's syndrome (a triad of jaundice, hemorrhage and acute renal failure), which has a 10–15% case fatality rate; and ii) severe pulmonary hemorrhage syndrome (SPHS), which may present as acute respiratory distress and was associated with case fatality rates >50% in several studies [7]–[10].

Prompt triage of high-risk patients is critical because complications require intensive care, specific treatment and monitoring. Although laboratory diagnosis is complex and requires specialized techniques such as microscopic agglutination technique (MAT) or real-time PCR, it is essential for biological confirmation.

Factors responsible for the manifestation of severe forms have not been clearly established [8], [11]–[18]. However, pathogen- as well as host-related factors are believed to play a role in the development of severe leptospirosis. This study aimed to determine the risk and prognostic factors independently associated with severe leptospirosis in laboratory-confirmed cases in adults in NC.

## Materials and Methods

### Study design

NC is an archipelago in the South Pacific with 249,000 inhabitants (Census 2009, New Caledonian Institute for Statistics and Economics, ISEE) and located approximately 1,200 km east of Australia and 1,500 km northwest of New Zealand. It comprises a main island (Grande Terre), the Loyalty Islands, the Isle of Pines, and several smaller islands. Fifty-two percent of the population lives in Noumea and its suburbs, 35% live in others districts on the main island and 13% live on the other islands.

We carried out an observational retrospective case-control study to explore the factors associated with severe leptospirosis among patients who were hospitalized with a biologically confirmed or probable leptospirosis between January 2008 and June 2011 in either of two public hospitals (Centre Hospitalier Territorial, Noumea and Centre Hospitalier du Nord, Koumac).

### Inclusion criteria

All patients hospitalized with a laboratory diagnosis of leptospirosis were retrospectively included. The enrollment criteria excluded patients who had no history of hospitalization, who were not residents of the study area and who were under 18 years old.

Biological diagnoses were performed at the Institut Pasteur of New Caledonia (IPNC). The MAT panel used for leptospirosis serology in New Caledonia was adapted to the local epidemiology and includes 11 pathogenic serovars and the Patoc strain, corresponding to the “local panel” described by Berlioz-Arthaud et al., 2007 [5]. Leptospirosis was categorized as either confirmed (patients who had: a positive PCR, a seroconversion from negative to a MAT titer ≥400 in paired serum samples or a fourfold increase between an acute serum sample and a convalescent serum sample using the reference MAT) or probable (patients who had both a clinical presentation of leptospirosis and a single MAT titer >400). Routine real-time PCR allowed the target organism to be quantified [19]. The sequence polymorphism of the *lfb1* gene diagnostic PCR products was used to identify the infecting strains [20], as this method was formerly demonstrated to allow identification of several different lfb1 sequence cluster types circulating in New Caledonia. MAT results in convalescent sera were also used to putatively identify the *Leptospira* serogroup [21].

### Case and control definitions

Cases were hospitalized patients who met the clinical definition for severe leptospirosis. Severe leptospirosis was defined by the presence of at least one of the following criteria: acute renal failure requiring dialysis, shock treated with vasoactive drugs, alveolar hemorrhage, bleeding requiring blood transfusion, respiratory insufficiency needing mechanical ventilation or death during hospitalization.

Controls were defined as patients hospitalized for milder forms of leptospirosis that presented none of these clinical complications, i.e. neither requiring dialysis, mechanical ventilation, blood transfusion or vasoactive drugs nor presenting an alveolar hemorrhage.

### Procedure and data collection

For each study participant, a standardized form was retrospectively completed.

Clinical manifestations and medical history, including current cigarette smoking, chronic alcoholism, respiratory insufficiency, diabetes mellitus and chronic hypertension were collected as mentioned in medical records. Demographic data and laboratory results were extracted from electronic records. All data were recorded with EPI Data 3.5.

### Ethics statement

The study was approved by the Clinical Research Committee of Institut Pasteur Paris and the research committee of Centre Hospitalier Territorial, Noumea. The study was also approved by the French consultative committee for the data processing in health research (Comité Consultatif sur le Traitement de l'Information en matière de Recherche dans le Domaine de la Santé, CCTIRS) and was authorised by the French Data Protection Authority (CNIL, Commission Nationale de l'Informatique et des Libertés). Informed oral consent was obtained from patients. The use of oral consent was approved by the CCTIRS because it was thought to be the most appropriate mode for this retrospective observational study. Oral consent was witnessed and documented on a form. All data analyzed were anonymized.

### Statistical analysis

Statistical analysis was performed using Stata 11 (StataCorp LP, College Station, TX, USA). Categorical variables were summarized using percentages and compared using the Chi-square test or Fisher's exact test. Continuous variables were summarized using means ± standard deviation (SD) and compared using Student's t-test or the Mann-Whitney test as appropriate.

Logistic regression was used to identify factors associated with severe leptospirosis and to estimate odds ratios (ORs) and 95% confidence intervals (CIs) for the associations between exposure variables and severe cases. Variables with p values<0.20 were introduced in the multivariate logistic regression model. A manual backward stepwise approach was used to remove non-significant variables; only variables with p values<0.05 were retained in the final model. Interactions were sought by introducing interaction terms in the logistic regression model and testing for their significance at the 0.05 level. The Hosmer-Lemeshow test was used to evaluate the goodness-of-fit of various models.

The biological variables that were included in the multivariate predictor model were comprised of missing values and resulted in losses of power and precision, complicated data handling and analysis, and potentially biased ORs due to differences between observed and unobserved data. We used multiple imputation (MI) to include all participants with missing responses in the analysis. Missing data were imputed using chained equations (ICE). The multiple imputation prediction model included all variables in the conceptual framework. Thirty imputed data sets were created and analyzed together. The critical thresholds we determined in this model were levels above or below those in which treatment decisions were usually made. Standard logistic regression models were fitted using STATA 11. The imputed data sets were analyzed in STATA 11 using the ICE suite of commands.

In addition, given the limitations imposed by the missing data, we performed a series of sensitivity analyses to assess the robustness of the analysis. In each case, we assumed a specific extreme scenario and apportioned missing values accordingly.

## Results

### Baseline characteristics

A total of 306 patients hospitalized with leptospirosis were diagnosed by IPNC between January 2008 and June 2011. One hundred and fifty of these cases were excluded from further analysis because they were not hospitalized in one of the two participating centers (n = 55), resided overseas (n = 4), were under 18 years old (n = 45) or could not be traced (n = 26).

Of the 176 patients included in the study, 152 (86.4%) were classified as confirmed leptospirosis and 24 (13.6%) were classified as probable leptospirosis (i.e., they had a clinical presentation of leptospirosis and a single MAT titer >400). The majority of study subjects were men (62.5%) with a mean age of 42.2±17.1 years. Subjects were mostly Melanesian (88.6%) living in tribes in rural areas (88.5%).

The frequency of clinical symptoms and complications are presented in Table 1. Jaundice, oliguria and conjonctival suffusion were more frequent among cases whereas the incidence of fever, myalgia and headache were not significantly different between cases and controls.

**Table 1:** Clinical symptoms in 176 leptospirosis patients in New Caledonia 2008–2011.

	Cases	Controls	
	N = 71	N = 105	P value
	n/N (%)	n/N (%)	
Baseline sign and symptoms			
Fever (>38°C) at admission	31/67 (46.3)	48/103 (46.6)	0.788
Myalgia	68/71 (95.8)	93/105 (88.6)	0.107
Headache	61/70 (87.1)	84/105 (80.0)	0.223
Jaundice	61/71 (85.9)	50/105 (47.6)	<0.001
Conjonctival suffusion	23/70 (32.9)	12/105 (11.4)	0.001
Oliguria	66/71 (93.0)	44/104 (42.3)	<0.001
Complications			
Acute renal failure with dialysis	23/71 (32.4)	—	NA
Pulmonary involvement needing mechanical ventilation	29/71 (41.4)	—	NA
Shock treated with vaso-active drugs	62/70 (88.6)	—	NA
Bleeding requiring blood transfusion	23/71 (32.4)	1/105 (1.0)*	NA
Alveolar hemorrhage	39/70 (55.7)	—	NA
Case-fatality rate	10/71 (14.1)	—	NA

* 1 patient had an already known chronic pancytopenia.

Seventy-one patients (40.3%) met our clinical definition for severe leptospirosis. Among them, 23 (32.4%) had acute renal failure requiring dialysis. Pulmonary involvement associated with mechanical ventilation was identified in 29 (41.4%) patients. Shock treated with vasoactive drug was documented in 62 (88.6%) patients, 23 (32.4%) patients received packed erythrocytes transfusion and 39 (55.7%) patients had alveolar hemorrhage.

Among severe cases, ten patients died and all had been admitted or transferred to the only intensive care unit in Noumea. The case fatality rate of the severe forms was 14.1% and the median time between admission and death was one day. Among the nine fatal cases for whom the serogroup was identified, seven were Icterohaemorrhagiae.

Both methods (MAT and PCR product sequence polymorphism) were used to identify the serogroup or the putative serogroup of the infecting strain in 161 patients (91.5%). Using MAT results, the serogroup involved was identified in 51 patients. Among these, 42 were confirmed cases with an acute and a convalescent serum and 9 had a single MAT titer ≥1600. The most common serogroups were Icterohaemorrhagiae observed in 37 patients, Australis in 6, Pyrogenes in 5 patients, Pomona in 1, Tarassovi in 1, Panama in 1.

The sequence polymorphism of lfb1 PCR products was used to identify the sequence cluster type of the infecting strains in 110 patients. The most common sequence cluster type identified was linked to serogroup Icterohaemorrhagiae in 76 patients, Pyrogenes in 21 patients, Ballum in 8 patients, Australis in 3, Pomona in 1 and Bataviae in 1.

For the 15 remaining cases, identification of the serogroup was not possible.

At the time of admission, 11 patients had acute co-infections with diseases such as laboratory confirmed dengue (n = 5), syphilis (n = 2), *Ascaris lumbricoides* infection (n = 1), *Chlamydia trachomatis* infection (n = 1), A (H1N1) pandemic influenza (n = 1) or tuberculosis (n = 1).

All patients tested positive for dengue were diagnosed by RT-PCR or NS1 antigen ELISA. Moreover, all 11 patients with co-infections were confirmed cases: the diagnosis of leptospirosis was confirmed by PCR in 10 and a seroconversion was ascertained in paired sera using the microagglutination test in one.

### Risk factors associated with severe leptospirosis

The factors associated with severe leptospirosis in the univariate analysis are presented in Tables 2 and 3. In a logistic regression model used to assess independent factors, current cigarette smoking (p = 0.003), *L. interrogans* from the Icterohaemorrhagiae serogroup (p = 0.011) and a delay of >2 days between the onset of symptoms and the initiation of antibiotherapy (p = 0.013) were independently associated with severe leptospirosis (Table 4).

**Table 2:** Demographic characteristics and medical histories of 176 leptospirosis patients in New Caledonia, 2008–2011.

		Cases	Controls		
		N = 71	N = 105	OR (95% CI)	P value^a^
		n/N (%)	n/N (%)		
Gender	Female	26/71 (36.6)	40/105 (38.1)	1	
	Male	45/71 (63.4)	65/105 (61.9)	0.94 (0.50–1.75)	0.84
Age (years)	Mean (SD)	42.6 (17.8)	41.6 (16.2)		
	[18–44]	44/71 (62.0)	66/105 (62.9)	1	
	[45–64]	21/71 (29.6)	23/105 (21.9)	1.37 (0.68–2.77)	
	[65–85]	6/71 (8.4)	16/105 (15.2)	0.56 (0.20–1.55)	0.29
Ethnic group	Melanesian	60/66 (90.9)	80/92 (87.0)	0.67 (0.24–1.88)	
	Other community	6/66 (9.1)	12/92 (87.0)	1	0.44
Living areas	Noumea	18/69 (26.1)	35/104 (33.7)	1	
	Bush	51/69 (73.9)	69/104 (66.3)	1.44 (0.73–2.82)	0.29
Current cigarette smoking	No	33/70 (47.1)	72/105 (68.6)	1	
	Yes	37/70 (52.9)	33/105 (31.4)	2.45 (1.31–4.57)	0.005
Chronic alcoholism	No	52/70 (74.3)	87/105 (82.9)	1	
	Yes	18/70 (25.0)	18/105 (17.1)	1.67 (0.80–3.50)	0.17
Respiratory insufficiency	No	68/70 (97.1)	96/105 (91.4)	1	
	Yes	2/70 (2.9)	9/105 (8.6)	0.31 (0.07–1.50)	0.15
Diabetes (type II)	No	62/70 (88.6)	95/105 (90.5)	1	
	Yes	8/70 (11.4)	10/105 (9.5)	1.22 (0.46–3.28)	0.68
Chronic hypertension	No	62/70 (88.6)	87/105 (82.9)	1	
	Yes	8/70 (11.4)	18/105 (17.1)	0.62 (0.26–1.53)	0.30

Abbreviations: OR, odds ratio; CI, confidence interval.

a Significant association was classified as P<.05.

**Table 3:** Characteristics of infections and the effects of therapeutic approaches among 176 leptospirosis patients in New Caledonia, 2008–2011.

	Cases	Controls		
	N = 71	N = 105	OR (95% CI)	P value^a^
	n/N (%)	n/N (%)		
Infecting serogroup				
Others	12/66 (18.2)	36/95 (37.9)	1	
*L. interrogans* serogroup Icterohaemorrhagiae	54/66 (81.8)	59/95 (62.1)	2.75 (1.30–5.82)	0.008
Duration of symptoms before admission to hospital (days)				
0–3	33/70 (47.1)	70/105 (66.7)	1	
>3	37/70 (52.9)	35/105 (33.3)	2.24 (1.21–4.17)	0.011
Delay between onset of symptoms and initiation of antibacterial therapy (days)				
0–2	18/69 (26.1)	46/102 (45.1)	1	
>2	51/69 (73.9)	56/102 (54.9)	2.33 (1.20–4.52)	0.013

Abbreviations: OR, odds ratio; CI, confidence interval.

a Significant association was classified as P<.05.

**Table 4:** Multivariate model of independent factors associated with severe leptospirosis (N = 156) in New Caledonia, 2008–2011.

	OR (95% CI)	P value^a^
Tabacco use	2.94 (1.45–5.96)	0.003
*L. interrogans* serogroup Icterohaemorrhagiae	2.79 (1.26–6.18)	0.011
Delay between onset of symptoms and initiation of antibacterial therapy >2 days	2.78 (1.31–5.91)	0.008

Abbreviations: OR, odds ratio; CI, confidence interval.

a Significant association was classified as P<.05.

### Prognostic factors associated with severe leptospirosis

The laboratory parameters at admission that were associated with severe leptospirosis are presented in Table 5 (before and after the MI procedure). In multivariate analysis, the following variables were linked with poor prognosis at referral: leptospiremia >10^3^ leptospires/mL before initiating treatment; platelet count ≤50×10^9^/L; serum creatinine >200 mM, serum lactate >2.5 mM and serum amylase >250 UI/L (Table 6).

**Table 5:** Initial laboratory findings among 176 leptospirosis patients in New Caledonia, 2008–2011.

		Cases	Controls		
		N = 71	N = 105	OR (95%CI)	OR (95%CI)
		n/N (%)	n/N (%)		MI procedure
Lactate (mM)	≤2.5	20/50 (40.0)	22/27 (81.5)	1	1
	>2.5	30/50 (60.0)	5/27 (18.5)	6.60 (2.15–20.31)	4.61 (3.77–5.63)
AST (UI/L)	≤150	48/66 (72.7)	87/97 (89.7)	1	1
	>150	18/66 (27.3)	10/97 (10.3)	3.26 (1.40–7.63)	3.15 (2.48–4.01)
Bilirubin (mg/dL)	≤50	28/66 (42.4)	77/94 (81.9)	1	1
	>50	38/66 (57.6)	17/94 (18.1)	6.15 (3.01–12.59)	6.09 (4.96–7.49)
Amylase (UI/L)	≤250	18/40 (45.0)	70/80 (87.5)	1	1
	>250	22/40 (55.0)	10/80 (12.5)	8.56 (3.44–21.24)	6.43 (5.19–7.97)
Creatinine (mM)	≤200	31/67 (46.3)	84/98 (85.7)	1	1
	>200	36/67 (53.7)	14/98 (14.3)	6.97 (3.32–14.63)	6.35 (5.13–7.86)
Hemoglobin (g/dL)	≤8	7/71 (9.9)	1/104 (1.0)	11.27 (1.35–93.70)	8.30 (4.74–14.55)
	>8	64/71 (90.1)	103/104 (99.0)	1	1
Leucocytes (G/L)	≤15	53/71 (74.7)	88/104 (84.6)	1	1
	>15	18/71 (25.3)	16/104 (15.4)	1.87 (0.88–3.97)	1.89 (1.50–2.37)
Platelet count (G/L)	≤50	31/71 (43.7)	94/104 (90.4)	12.13 (5.43–27.08)	12.12 (9.52–15.43)
	>50	40/71(56.3)	10/104 (9.6)	1	1
Prothrombin time (%)	≤70	21/57 (36.8)	20/76 (26.3)	1.63 (0.78–3.43)	1.70 (1.40–2.07)
	>70	36/57 (63.2)	56/76 (73.7)	1	1
Leptospiremia*	≤1000	22/35 (62.9)	44/47 (93.6)	1	1
	>1000	13/35 (37.1)	3/47 (6.4)	8.67 (2.23–33.6)	2.33 (1.90–2.87)

Abbreviations: OR, odds ratio; CI, confidence interval; MI, multiple imputation.

**Table 6:** Multivariate model of independent biological factors associated with severe leptospirosis (N = 176) in New Caledonia, 2008–2011.

	OR (95% CI)
	MI procedure
Platelet count ≤50 (G/L)	6.36 (1.79–22.62)
Creatinine >200 (mM)	5.86 (1.61–21.27)
Lactate >2.5 (mM)	5.14 (1.57–16.87)
Amylase >250 (UI/L)	4.66 (1.39–15.69)
Leptospiremia >1000 (leptospires/mL)	4.31 (1.17–15.92)

Abbreviations: OR, odds ratio; CI, confidence interval; MI, multiple imputation.

To explore the effects of potential biases related to the missing values in the model including all the patients, we tested the sensitivity of our multivariate model to a series of assumptions about the missing values. Analysis of the associations between most of these parameters and severe leptospirosis resulted in similar OR estimates regardless of the hypothesis used to account for the missing values.

## Discussion

This retrospective case-control study of leptospirosis in NC allowed us to identify risk factors associated with severe forms of leptospirosis in adults. In parallel, we also identified biological variables evident at the time of admission that were predictors of poor outcomes.

Our main findings indicated that the *L. interrogans* serogroup Icterohaemorrhagiae was significantly associated with severe forms of leptospirosis. Multivariate analysis identified other risk factors linked to the host or to the initial management of the disease: smoking was an independent factor of severity, particularly in the pulmonary involvement of the disease. Also, if antibiotics were started later than two days after symptom onset, the risk of developing a severe form was considerably higher than if antibiotics were started earlier.

The first laboratory findings available after admission revealed certain parameters that correlated strongly with severe outcomes. Leptospiremia higher than 10^3^ leptospires/mL before treatment was initiated was associated with a higher risk of developing severe leptospirosis. Acute renal failure, acute pancreatitis, a low platelet count and a high serum lactate level at admission were also indicators of a poor prognosis.

We classified the severity of leptospirosis based on clinical criteria, which was a pragmatic approach meant to reflect the routine management of patients. We focused on hospitalized patients that had a laboratory diagnosis of leptospirosis. Nevertheless, determining the case definition was difficult, since there is no consensus for severe forms in leptospirosis. Classically, the known severe forms are characterized by Weil's syndrome. The emergence of SPHS has been described more recently [8]–[10]. Thus, some studies using similar methodologies have defined severe cases as those involving patients with SPHS as opposed to patients with non-severe pulmonary forms [8], [12], [22]. Other authors restricted the definition of severe forms to fatal outcomes during hospitalization [14], [15], [23].

Another option was to classify patients hospitalized for leptospirosis in intensive care units as severe cases and patients hospitalized for leptospirosis in other hospital departments as controls [12]. However, this definition does not take into account the patient's clinical condition at admission and could have been biased because of the patient's distance to the only ICU in Noumea. Similar to our classification, Herrmann-Storck et al. [11] used treatment-related criteria, such as the need for hemodialysis or mechanical ventilation, to define severe cases in Guadeloupe and Spichler et al. also used clinical signs to define severity [24].

One major finding of our study was the independent association between the Icterohaemorrhagiae serogroup and severe leptospirosis in the multivariate analysis. Though Icterohaemorrhagiae has been previously suspected to cause more severe forms of the disease, this relationship has been seldom demonstrated in a significant number of patients [21]. Additionally, sequence-derived identification of the infecting strain provides a higher degree of confidence than MAT-derived putative identification of the serogroup, especially at the individual level [21]. Our identification of the infecting strain in patients diagnosed by PCR increased the statistical power of our analysis and provided a larger set of data. Several hypotheses regarding leptospirosis severity are based on host genetic susceptibility factors [25], [26] and/or on bacterial virulence [27], although the virulence mechanisms are poorly understood and are probably multifactorial [28]. In NC, rodents are the main reservoir of leptospirosis. Three rat species (*Rattus rattus*, *Rattus exulans* and *Rattus norvegicus*) introduced during different periods of settlement contribute to the maintenance and transmission of the Icterohaemorrhagiae serogroup [3], [29], [30]. Our results demonstrating this strain's high pathogenicity highlight the importance of controlling rodent populations, not only to lower the risk of transmission, but also to limit the number of severe forms.

In a retrospective study in 17 patients with pulmonary involvement, Martinez Garcia et al. found that smoking was a risk factor for respiratory involvement in human leptospirosis [31]. Our multivariate analysis is in accordance with this finding: smokers were three times more likely to develop severe leptospirosis than non-smokers, and the risks of alveolar hemorrhage and respiratory distress in smokers were especially high. It is suggested that some tobacco components can favor the development of pulmonary haemorrhage by increasing the permeability of lung capillaries, damaging alveolar basement membrane and increasing the local inflammatory response [32]. This association with smoking has also been found for varicella pneumonia [33].

Another noteworthy finding was that the delay before antibacterial therapy had a major impact on outcome. This finding, which agrees with previous reports [11], [16], illustrates the need for early initiation of antimicrobial therapy to reduce disease severity. Presumptive treatment based on clinical and epidemiological evidence therefore appears justified while waiting for the laboratory results. In addition, leptospires are usually susceptible to most common antibiotics [34] and the daily treatment costs for standard regimens of beta-lactam/cephalosporins or tetracyclines are low.

Few reports have described underlying conditions that are significantly associated with the severity of acute leptospirosis. Although chronic alcoholism and hypertension have been associated with severity [11], we did not confirm this in our multivariate analysis.

Consistent with some previous studies, our results suggest that there is no relationship between gender and the severity of the disease [11], [14]–[16], [22], [23]. Previous reports have been controversial about this factor: only two studies have shown that the male sex was independently linked to severe disease [8], [35]. The sex ratio in our study population was 1.7 male/female, which agrees with the leptospirosis incidence typically found in NC [5]. Numerous studies have described age as a predictor of death [14], [15], [23], [24], [35]. Our study, in accordance with Gouveia et al. did not identify age as a risk factor [8].

In agreement with previous reports [11], [12], [15], we identified laboratory parameters at the time of admission that were predictive of severe forms. The critical thresholds we determined in this model were above or below the levels at which treatment decisions are usually made. Early determination of these parameters could provide an alert to guide physicians in their management of patients at high risks.

Quantification of leptospires in clinical samples could have a prognostic value if performed routinely. Segura et al. described high levels of leptospires in the lung, liver, muscle and kidney tissues of patients who died and found that the presence of at least 10^4^ leptospires/mL of blood can be considered a critical threshold for fatality [36]. These findings agreed with those of Truccolo et al. [37].

We considered it relevant to define a leptospiremia threshold associated with disease severity. In our study we determined the leptospiremia in patients who had been diagnosed by quantitative PCR, but we only kept the values for blood samples taken before antibacterial therapy was started. This forced us to exclude data if the exact date of sampling was not available, and led to a substantial number of missing values in our model. We therefore performed a MI analysis, which is a well-established method for analyzing data sets with missing values, and showed by multivariate analysis that a threshold of 10^3^ leptospires/mL of serum strongly correlated with severe forms. In a recent study, leptospiremia did not correlate with clinical manifestations of outcome [38]. To our knowledge, our study is the first one that clearly delineates an association between leptospiremia levels and disease severity.

One limitation of the present study is its retrospective design. Indeed, it was sometimes difficult to estimate longitudinal parameters, such as the delay between the blood sampling for laboratory diagnosis and the initiation of the antibacterial therapy. We therefore used MI to account for the missing values and assumed that the data were not missing completely at random. Additionally, we performed a sensitivity analysis to explore which effects might have biased our results. Even under the most extreme hypothesis in the sensitivity analysis, the same trends were evident.

We also distinguished the risk factors and the prognostic factors associated with severe leptospirosis, as presenting them in two separate models may be informative. Physicians cannot intervene with risk factors directly but they can identify high-risk patients. On the other hand, prognostic factors occur secondary to the infection, so taking them into account early and initiating appropriate treatments may limit the development of severe leptospirosis.

In conclusion, leptospirosis is responsible for a high number of hospitalizations due to severe forms of the disease in NC. To prevent and control this public health threat, some recommendations may be inferred from our results. The predominance of the Icterohaemorrhagiae serogroup, which independently associated with severe forms of leptospirosis, highlights the possible benefits of rodent control measures. We support the advantages of early antimicrobial therapy initiation in suggestive epidemiological and clinical contexts, even before laboratory confirmation of leptospirosis. As reported previously, cigarette smoking was more common in severe leptospirosis than in mild forms. Physicians should focus on the pulmonary function of their patients, especially if they are smokers. To improve the early management of high-risk patients, some laboratory criteria could be used at the time of admission. High creatinine, lactate and amylase levels and low platelet counts were associated with severe leptospirosis. Our data illustrate the benefit of using a critical threshold of qPCR-determined leptospiremia to assess the risk of developing severe leptospirosis. Leptospirosis remains a major medical challenge, especially in tropical areas and particularly for severe forms that can progress rapidly to multiple organ failure. Physicians need to be aware of the factors associated with severe leptospirosis to reduce severity and mortality through the timely management of patients.

## Supporting Information

Checklist S1 **STROBE checklist.**
(DOC)
